# Why Having a (Nonfinancial) Interest Is Not a Conflict of Interest

**DOI:** 10.1371/journal.pbio.2001221

**Published:** 2016-12-21

**Authors:** Lisa A. Bero, Quinn Grundy

**Affiliations:** Charles Perkins Centre, Faculty of Pharmacy, University of Sydney, Sydney, Australia

## Abstract

A current debate about conflicts of interest related to biomedical research is to question whether the focus on financial conflicts of interest overshadows “nonfinancial” interests that could put scientific judgment at equal or greater risk of bias. There is substantial evidence that financial conflicts of interest such as commercial sponsorship of research and investigators lead to systematic biases in scientific research at all stages of the research process. Conflation of “conflicts of interest” with “interests” in general serves to muddy the waters about how to manage conflicts of interest. We call for heightened disclosure of conflicts of interest and policy action beyond disclosure as the sole management strategy. We propose a different strategy to manage interests more broadly to ensure fair representation and accountability.

In 2005, a Scottish judge ruled against the plaintiff, a lifelong smoker, in *McTear v*. *Imperial Tobacco Limited* [1]. In part of his judgment, Lord Nimmo Smith contrasted the expert witnesses handsomely paid by Imperial Tobacco with the plaintiff’s unpaid experts. He contended that the paid expert witnesses were less biased than the unpaid experts who had dedicated their lives and professional careers to their scientific research. In this case, the judge believed that the experts’ “nonfinancial” interests constituted the greater risk to expert opinion.

A current debate about conflicts of interest related to biomedical research is to question whether the focus on financial conflicts of interest overlooks “nonfinancial” interests that could put scientific judgment at equal or greater risk of bias [2–5]. As shown in Box 1, these important and influential nonfinancial interests range from personal beliefs to past experience to theoretical commitments to the desire for enhanced reputation and career advancement [6]. Many scientific organizations have aimed to identify these “nonfinancial” interests by appending a laundry list to standardized conflict of interest disclosure forms. For example, a recent cross-sectional study found that 57% of the National Library of Medicine’s core clinical journals required disclosure of at least one form of “non-financial conflict of interest” [6].

**Box 1. Examples of Interests in Biomedical Research**

Personal, religious, or political beliefs

Personal experiences

Advocacy or policy positions of the researcher or organization with which they are affiliated

Intellectual, theoretical, or school of thought commitments

Type of training; professional or academic education

Profession or discipline

Academic competition or rivalry

Career advancement or promotion

Glory seeking or desire for fame

Dominant researcher in area of research

Personal experience with subject of the research

Personal relationship with someone who has the disease or condition under study

Role as investigator on study included in a systematic review

Published opinion essay or commentary on topic of research

Institutional affiliation or academic associations

There is substantial evidence that financial conflicts of interest such as commercial sponsorship of research and investigators lead to systematic biases in scientific research at all stages of the research process [7–11]. Focusing on interests such as personal beliefs, experience, or intellectual commitments can divert attention from financial conflicts of interest, which have the potential for widespread influence. The result is an erosion of the evidence base and confidence in science, making it vulnerable to competing groups’ claims [12], as we are seeing with issues as diverse as childhood vaccination and climate change.

The concern over “nonfinancial” interests is in part due to the growing recognition that the social context in which research is conducted will impact the research questions that are asked, the studies that are funded, the research outcomes, their dissemination, and their interpretation. Scientists have long operated under the pretext that they can and should be completely neutral when approaching the research process, without any desire to influence the outcome. Yet, social values, interpretation, and theoretical commitments are essential parts of research at every phase [13]. When past experiences, personal identities, or expertise are treated as conflicts of interest, individuals with a stake in the research process are excluded. At the same time, those that claim to be “neutral”—though they too have a particular stake in the process—attain a privileged position, and their interests are allowed to shape the research.

Conflation of “conflicts of interest” with “interests” in general serves to muddy the waters about how to manage conflicts of interest, generating confusion as to the nature and definition of the problem and doubt as to whether conflicts of interest can be addressed at all [14]. Taking the “laundry list” approach, in which anything and everything becomes labelled a “conflict of interest,” will not be effective in managing the influence of interests more broadly. In this paper, we clarify the concept of conflict of interest. We call for heightened disclosure of conflicts of interest and policy action beyond disclosure as the sole management strategy. The scientific community requires new tools to account for the influence of identities and interests in research. We adopt a multidisciplinary perspective and borrow from the social sciences to propose the use of reflexivity, a tool for identifying and engaging such interests.

## Refocusing Definitions of Conflict of Interest

The Institute of Medicine (IOM) defines a conflict of interest as “a set of circumstances that creates a risk that professional judgment or actions regarding a primary interest will be unduly influenced by a secondary interest” [15]. Conflicts of interest are a problem for those who must make expert judgments on behalf of others; thus, their primary interest is the well-being of those who rely upon these judgments. For researchers, this is the scientific community and public, who make decisions on the basis of the outcomes of research. Conflicts of interest are distinct from ethical dilemmas in that one interest has a claim to priority—the primary or professional interest—and efforts are directed at ensuring that secondary interests do not dominate, or appear to dominate, the primary interest [14].

Being a researcher means having particular education and training, typically a personal interest in a topic or field, usually employment with some kind of academic or scientific institution, and a whole host of experiences that make up a research career. These interests are part of the primary roles and responsibilities that come with being a scientist [13]. However, interests are distinct from conflicts of interest (Box 2).

**Box 2. Is an Interest a Conflict of Interest?**

- The individual or institution is in a position where others rely on their decision making.
- One of the conflicting interests has an ethical claim to priority.
- It is theoretically possible to eliminate the conflict of interest. (If the only solution is recusal, this is not a conflict of interest.)
- The direction of bias produced by the conflict of interest is consistent within a set of circumstances.
- The scope of influence may extend beyond an individual and immediate set of circumstances, as in the case of sponsorship.

We propose three rules of thumb to distinguish conflicts of interest from “interests” more broadly. First, it is theoretically possible, though not always necessary, to eliminate a conflict of interest. For example, an investigator can divest themselves from shares in the company that commercializes their research product, whereas they cannot possibly separate themselves from their disciplinary training. Similarly, if the only solution for a particular type of interest is recusal because the interest cannot be eliminated, this is not a conflict of interest but rather part of the researcher’s professional role or personal identity.

Second, the direction of the bias produced by a conflict of interest is consistent within a set of circumstances. Evidence examining the influence of industry sponsorship on research suggests that the existence of these conflicts may systematically distort the outcomes, effect sizes, or conclusions of research in a direction which consistently favors the sponsor [7–11].

Third, conflicts of interest can be widespread, and their scope of influence may extend far beyond an individual. People participate in research as individuals, and each bring their experiences and personal and professional interests to the process. However, sponsorship serves to amplify a particular viewpoint, ensuring its widespread dissemination and representation in decision making. For example, corporate sponsorship of research is capable of distorting an entire body of published research, as was the case with the tobacco industry and research on secondhand smoke [16].

Conflicts of interest are not exclusively financial: for example, conflicts of interest can arise from personal relationships. First, a person theoretically can eliminate the conflict by eliminating the relationship (which in most cases is an extreme measure) or, more commonly, by reorganizing roles and responsibilities or seeking oversight. For example, spouses working in the same lab commonly require special permission from the institution and are not allowed to directly supervise each other. Recusal is a common strategy but, importantly, not the only option. For example, individuals disclose the relationship and typically recuse themselves from reviewing grant applications written by their students, postdocs, collaborators, rivals, or family members. Second, the direction of the bias can almost always be predicted with conflicts of interest arising from personal relationships—individuals will typically help their friends and harm their enemies. However, while personal relationships can lead to conflicts of interest, unlike conflicts of interest arising from financial ties, their effects rarely extend beyond the immediate situation. That said, conflicts of interest stemming from relationships may be more damaging for individuals in positions of power (such as supervisory or leadership roles), as their scope of influence is greater.

## Why Financial Conflicts of Interest are so Problematic

Commercial sponsorship of research and investigator financial conflicts of interest are two forms of conflict of interest with the strongest evidence base, suggesting that they are a widespread and harmful source of bias. Pharmaceutical, tobacco, food, or chemical industry funding biases human research studies towards outcomes that are favorable to the sponsor’s product, even when controlling for other biases in the methods [7–8,10–11,17]. Thus, even when the methods meet high standards for internal validity, financial conflicts of interest may influence research results through other mechanisms, such as the framing of the question, how the study is actually conducted, and whether it is fully and accurately reported.

While everyone comes to the table with an identity, past experiences, and professional interests, industry sponsorship or investigator payments serve as a megaphone, amplifying and multiplying a set of interests that align with the sponsor’s and thereby create a widespread platform of influence for the sponsor. The tobacco industry for decades used sponsorship of research and payments to scientists to fund research that supported their interests, to suppress research that did not, and to disseminate interest group data to the lay press and policymakers [16]. The influence of industry sponsorship on research has created a crisis within biomedicine—not only of confidence in the evidence that underlies clinical practice and public policy, but the integrity of the research itself. For example, Coca-Cola’s sponsorship of public health research resulted in the reshaping of an entire field of research to focus on physical activity to reduce obesity, to the exclusion of nutrition-related research [18].

## Not All Interests are Conflicts of Interest

The growing concern about “nonfinancial” interests may be part of the recognition that the social context in which research is conducted influences the results. For example, researchers conducted replications of 100 experimental and correlational studies in psychology and found that over half of the replications produced weaker evidence for the original findings, despite attempts to use the author’s original materials [19]. This suggests that something other than the research design produced such variation. In science, there has been a common pretext that the researcher should approach the research without any desire to influence the outcome and that any conclusions should be driven solely by the data. Social scientists, however, have long argued that it is not possible to be impartial, disinterested, or value-neutral and that it is essential to acknowledge this as a means of being answerable for what science claims to know about the world [20–21]. Feminist scholars in particular have argued that it is not possible to be neutral in research as it is neither possible nor even desirable for scientists to be interest-free [21–22]. While it is essential to systematically examine all of the social values shaping a research process, these cannot possibly be eliminated but must instead be made visible and open to critical interpretation [23]. However, this does not mean that one cannot be objective or that all interests and values are merely relative. Instead, scholars have advocated for a “passionate detachment,” which emphasizes fairness, honesty, and recognition of the social positions and interests that influence how evidence is produced and shared [20–21].

Recognizing that scientists possess an interested view—grounded in personal experiences and beliefs, education and disciplinary training, and intellectual commitments—is crucial to rigorous research and healthy scientific debate, but it is distinct from conflict of interest. Scientists cannot be separated from their interests or their social position in the world. Recusal of scientists based on their personal beliefs and experience can serve exclusionary purposes and falsely identify certain individuals, who also possess personal beliefs and experiences, as “objective,” narrowing the diversity of perspectives involved in decision making. For example, a recent analysis of recusals from Food and Drug Administration (FDA) advisory committees on the basis of “intellectual conflicts of interest” found that each instance led to a decision that favored industry interests and that no expert had been excluded because he or she supported a particular drug or device [24]. Similarly, the effect of interests on research is isolated to the sphere of influence of the individual, and the direction of the bias created cannot be predicted. A scientist seeking academic glory may do so on either side of a research agenda—for example, climate scientists and climate change-deniers have achieved equal renown. It could be argued that researchers’ personal and intellectual interests are fundamental to good research and that these interests provide the momentum for scientific ideas to persist long enough to be scrutinized and, ultimately, to be useful.

## Managing Interests and Dealing with Conflicts

Confusing “interests” with “conflicts of interests” makes conflicts of interest appear so pervasive that they cannot be avoided but only disclosed. Rather, there are precedents for disclosing and managing financial conflicts of interest, which should be adopted and enforced widely within biomedical research institutions.

Disclosure is only a first step: requirements for disclosing financial conflicts of interest should be enforced, clear, and should list examples of financial conflicts of interest, following the example of the ICMJE [25]. Most disclosure policies currently rely on self-report; however, regulation such as the United States Physician Payments Sunshine Act could be expanded to include researchers and institutions to provide a more comprehensive source of data on financial conflicts of interest. While disclosure is essential in order to understand the scope of the problem and its effects, it is necessary that policy action go beyond disclosure to ensure that disclosures do not provide a moral license to dispense with their actual management [26].

Organizations, such as research institutions, scientific journals, and grant review panels should have policies for reviewing, mitigating, or eliminating financial conflicts of interest, and these should be enforced. Before engaging with a commercial entity, the proposed interaction should be subject to a thorough risk–benefit assessment by the research institution that includes the risk for reputational damage or loss of trust in the research activity [27]. For example, the Charles Perkins Centre at the University of Sydney has guidelines that require a committee to assess the alignment of a sponsor with the Centre’s mission; potential influence on the design, conduct, and publication of research; and the reputational risk to the institution [27]. Researchers should not engage with industry when the commercial interests are not aligned with improved public health or when the sponsor has any control over the design, conduct, or dissemination of the project [27]. Scientific institutions should consider prohibiting the acceptance of funding or publication of research when industry sponsorship or investigator conflicts of interest pose too high a risk. For example, several biomedical journals will not publish tobacco industry–funded research [28], and some universities do not accept tobacco industry funding for research [29].

The biomedical community needs new tools to account for the influence of interests and identities more generally on research. Reflexivity is a tool that could be borrowed from the social sciences and adapted to biomedical research that makes transparent and accounts for researchers’ professional and personal identities. It can be implemented at the level of the individual scientist but perhaps can be more effective when built into existing institutional processes. For example, if a university wished to set up a center on reproductive technology using CRISPR to genetically modify embryos, the steering committee would be well served to identify personal interests and past experience with the issue. Experts and those with particular opinions on the matter would not be recused, but perhaps opposing perspectives would be purposely included and processes put in place to ensure fair representation and an evidence-informed approach [30]. In lab settings, reflexivity has been used to make day-to-day research decisions transparent and to evaluate these decisions in terms of the interests at play [31–32]. Box 3 outlines key questions that are characteristic of reflexive processes [33].

**Box 3. Key Questions for Reflexivity [33]**

Who is the researcher?

- What are their *professional* identities? What is their discipline, educational background, or training? Where are they employed? What is their career stage, and are they in a position of power or influence? What is their area of research or theoretical perspective? What are their advocacy positions?
- What are their relevant *personal* identities, including age, race/ethnicity, gender, religious or political affiliations, and life experience?

How could who they are affect the design, conduct, or reporting of research?

Who or what is the focus of the research? For whom does this have consequences? What are these consequences?

Who or what is placed at risk by this research? How?

Who or what is advantaged by this research? How?

What are the ethical, social, political, or economic implications of this research?

We use a grant funding review panel as an example in Table 1 to show how two parallel processes can be used simultaneously: reflexive processes to address relevant interests and comprehensive disclosure and management for conflicts of interest (Table 1). These practices could also be useful at the research agenda–setting phase, in determining funding priorities, or in peer review processes. The goal is to hold researchers accountable without discrediting scientific findings and claims as mere “personal biases” [33] and to ensure fair representation rather than excluding certain individuals based on their personal characteristics, beliefs, or expertise.

**Table 1:** A grant proposal review panel: a hypothetical example of reflexivity and conflict of interest management.

Phase	Nonfinancial interests	Conflicts of interest
Panel formation	Prospective panel members are approached, aiming for diversity in terms of education, discipline, gender, and race/ethnicity, with a focus on contrasting opinions and representation from those both within the field of research and external perspectives. Prospective panel members write a narrative position statement, which addresses key reflexive points: Biographical details Relevant personal details (at individual’s discretion) Reflection on how who they are might influence the funding process Statement of purpose and interest in participating	Potential panel members are asked to report any financial conflicts of interest using a structured disclosure form. Disclosures of potential panel members are supplemented with data on financial conflicts of interest using a database such as Open Payments
Panel selection	Position statements are reviewed for: What perspectives are represented? Are any positions over represented? Under represented? Panel is reselected accordingly, aiming for diversity.	Panel chair without any financial conflicts of interest is selected. A panel is selected in which less than half of the members have financial conflicts of interest.
Panel deliberations	A reflexive process such as deliberative dialogue [34–35] is implemented, facilitated by the chair.	Panel members with relevant financial conflicts of interest are recused from discussion and final decision making regarding relevant applications.
Funding decisions and dissemination of results	A narrative summary of the panel members’ position statements is published to discuss how the panel composition influenced the funding outcomes. A summary of the review process reflects on and takes accountability for: Who and/or what is the target of the funded research? With what consequences? For whom? Who and/or what is placed at risk by the funded research? How? Who and/or what is advantaged? How?	All financial conflicts of interest and how they were managed are reported.

## Conclusion

The muddying of the waters around conflicts of interest serves to erode the political will to identify and manage conflicts of interest in scientific research, as they are portrayed as increasingly ubiquitous and unsolvable. A substantial evidence base supports the need for continued policy action, particularly around the disclosure and management of commercial sponsorship of research and investigator financial conflicts of interest. Different strategies are needed to manage interests more broadly to ensure fair representation and accountability.
